# Comparison of the effect of marine-derived omega-3 fatty acids (n-3 FAs) as an adjunct to a non-steroidal inflammatory drug (NSAID) therapy vs NSAID therapy alone, for dogs with osteoarthritis

**DOI:** 10.18849/ve.v7i1.527

**Published:** 2022-01-12

**Authors:** Lok Yee Stephanie Wong+, Merran Govendir+

**Keywords:** CANINE, FATTY ACIDS, JOINT SUPPLEMENTATION, OMEGA-3, OSTEOARTHRITIS, N-3 FAS, MON-STEROID ANTI-INFLAMMATORY DRUGS, NSAID, NUTRACEUTICALS

## Abstract

PICO question

Does treatment with a non-steroidal anti-inflammatory drug (NSAID) with supplementation of marine-derived  omega-3 fatty acids (n-3FAs) compared to the NSAID alone, result in an increased ability to exert force by the osteoarthritic limb(s) of dogs or alleviate other measures of osteoarthritis?

Clinical bottom line

Category of research question

Treatment

The number and type of study designs reviewed

Two prospective, block-randomised, clinical trials

Strength of evidence

None

Outcomes reported

Kwananocha et al. (2016) investigated administration of carprofen supplemented with marine-derived n-3 FAs, to carprofen alone, administered over 4 weeks. Vijarnsorn et al. (2019) investigated administration of firocoxib supplemented with n-3FA, to firocoxib alone, for 4 weeks.  There were no statistical differences between treatment groups at week 2 and week 4 post-treatment for either study. Both studies also reported orthopaedic assessment score (OAS) based on scoring the extent of patient lameness and pain in the affected joint. There were no statistical changes in OASs between treatment groups at week 2 or week 4 post-treatment for either study

Conclusion

There is no evidence that marine-derived n-3 FAs provide additional benefit when used as adjunctive agents with NSAIDs for the treatment of canine osteoarthritis

## How to apply this evidence in practice

The application of evidence into practice should take into account multiple factors, not limited to: individual clinical expertise, patient’s circumstances and owners’ values, country, location or clinic where you work, the individual case in front of you, the availability of therapies and resources.

Knowledge Summaries are a resource to help reinforce or inform decision making. They do not override the responsibility or judgement of the practitioner to do what is best for the animal in their care.

## Clinical scenario

A 4 year old Rottweiler (male, neutered) is being treated with NSAIDs to manage osteoarthritis (OA) of the hip joints. The owners want to try omega-3 fatty acids (n-3 FAs) supplementation, and you (the clinician) are unaware about the evidence-basis for this.

## The evidence

Two prospective, block-randomised clinical trials were found to match the PICO. Both studies assessed the effects of administering the same n-3 FAs with NSAID therapy compared to NSAID therapy alone. The n-3 FAs was a green-lipped mussel extract referred to as PCSO-524 marketed as Antinol® (Pharmalink International Ltd [Hong Kong]). Pharmalink provided financial support to both studies, and the same investigators performed both studies.

Both studies recruited canine hospital outpatients with a diagnosis of OA. Patients were randomly assigned to treatment groups. One treatment group was treated with a veterinary registered NSAID, and another was treated with the same veterinary registered NSAID and n-3 FAs. Both groups were administered their respective medications twice daily for 4 weeks. Patients underwent force plate gait analysis, gait observation and an orthopaedic examination that resulted in an OAS (Moreau et al., 2003) at week 0, and week 2 and week 4 post-treatment (i.e. weeks 6 and 8 of both studies).

Both studies found no significant benefit in the supplementation of n-3 FAs with NSAIDs, versus NSAIDs alone with respect to increasing the peak vertical force (PVF) generated by the arthritic limb, nor any improvement in the OAS. There are study design limitations in both studies such as inadequate sample size, and other factors such as an insufficient dosage, insufficient duration of dosage and brief study duration.

## Summary of the evidence

**Kwananocha et al. (2016):** 

Population:	Dogs from a client-owned referral population with a history of hindlimb lameness and radiographic changes consistent with hip and/or stifle OA older than 2 years old and within a bodyweight range of 18–50 kg.
Sample size:	40 dogs (10 dogs in each group). 49 dogs were initially enrolled, and nine were lost to the study or dismissed.
Intervention details:	Before the start of the study: Washout period of 2 weeks for NSAIDs and oral nutraceuticals. Washout period of 4 weeks for corticosteroids and injectable sodium-pentosan polysulphate. Dogs were examined and classified into two categories based on the subjective OAS system (Moreau et al., 2003) to have: Mild/moderate OA; Severe OA. Allocation of treatment groups at the start of the study (week 0): *Perna canaliculus* Lipid Complex (PCSO-524®) (10 dogs). Each PCSO-524® capsule contained: PCSO-524® (50 mg); Olive oil (100 mg); d-Alpha-tocopherol (0.225 mg); Dosage: two capsules, q12hrs PO, for 4 weeks. Glucosamine and avocado/soybean unsaponifiables (marketed as Dasuquin® by Nutramax Laboratories Veterinary Sciences, Inc. [USA]) group (10 dogs); Dosage: one tablet, q12hrs PO for 4 weeks. Carprofen group (10 dogs); Dosage = 2.2 mg/kg, q12hrs PO for 4 weeks. Combination group (10 dogs); Dosage = carprofen: 2.2mg/kg, q12hrs PO; and PCSO-524®: two capsules, q12hrs PO; both for 4 weeks. Only the carprofen and combination treatment groups are relevant to the PICO question. Force plate gait analysis to obtain peak vertical force (PVF) values: A single handler led a dog at a trotting pace along a 10 m walkway embedded with dual force plates to obtain a minimum of four valid PVF values. Force plate gait analysis was performed at weeks 0, 6 (2 weeks post-treatment) and 8 (4 weeks post-treatment). OAS: An orthopaedic examination was performed by a single veterinarian who was blinded to the treatment assignment for each patient. Scores for clinical parameters (lameness, articular pain for the hip, and stifle joint) were tallied. OAS was performed at weeks 0, 6 (2 weeks post-treatment) and 8 (4 weeks post-treatment).
Study design:	Prospective, block-randomised, single-blinded, clinical trial.
Outcome studied:	PVF and OAS.
Main Findings (relevant to PICO question)	PVF results: No significant treatment effects between treatment groups at week 0. Significant effects of time were found within treatment groups: When comparing week 6 to week 0, PVF values for the carprofen group and combination group were significantly greater with mean ± SD change 2.58 ± 2.48 % body weight (BW) (p = 0.028) and 4.39 ± 2.56 % BW (p = 0.001), respectively. Comparison of week 8 vs week 0, PVF values for carprofen group and combination group were significantly increased with mean ± SD change 4.23 ± 2.33 % BW (p = 0.001) and 5.36 ± 2.98 % BW (p = 0.001), respectively. Comparison of week 8 and 6 to week 0, there were no significant changes in PVF values between groups. OAS results: No significant treatment effects between treatment groups at week 0. Significant time effects were found within treatment groups: When comparing week 8 to week 0, OAS for the carprofen and combination groups were significantly greater (p = 0.048 and 0.029, respectively). Comparison of week 8 and 6 to week 0, there were no significant changes in OAS values between groups.
Limitations:	Details regarding the classification of dogs into mild/moderate or severe OA were not provided. It was unclear which limb was to be followed for force plate gait analysis in dogs with bilateral OA. The process of randomisation was not specified. The conditions in which dogs were managed during the study period were unknown. A delay in evaluating outcomes at 2- and 4-weeks post-treatment may have also allowed for the exacerbation of any variation, possibly impacting the validity of the results. Study period (treatment duration = 4 weeks) may have been too short to detect significant differences (Zawadzki et al., 2013). Analysis of variables at pretreatment (week 0), body condition score was borderline significant (p = 0.55). This may be a confounder, and it is possible that end-of-treatment results are not solely due to the intervention (Pannucci & Wilkins, 2010). No sample size calculation was reported. The repeatability of the study may be limited by the lack of detail in the methodology. PVF and OAS are surrogate outcomes and may not accurately reflect if the patient's mobility has improved or pain has reduced (Administration USFaD, n.d.).

**Vijarnsorn et al. (2019):** 

Population:	Dogs from a client-owned referral population with a history of chronic OA of the hip and/or stifle joints (hindlimb lameness and joint pain) and consistent radiographic changes, older than 1 year old, and a bodyweight of at least 20 kg.
Sample size:	79 dogs. Initially, 82 dogs were enrolled, but three were lost to the study or dismissed.
Intervention details:	Before the start of the study: A washout period of 2 weeks for NSAIDs and oral nutraceuticals. A washout period of 4 weeks for corticosteroids and injectable sodium-pentosan polysulphate. A single veterinarian examined and classified all dogs into two categories based on the subjective orthopaedic assessment scoring (OAS) system (Moreau et al., 2003): Mild/moderate OA; Severe OA. Allocation of treatment groups at the start of the study (week 0): Firocoxib group (24 dogs); Dosage: firocoxib: 5 mg/kg, q24hrs PO; and PCSO-524® placebo: 4 capsules/day, q24hrs PO for 4 weeks. PCSO-524® group (27 dogs). Each PCSO-524® capsule contains: PCSO-524® (50 mg); Olive oil (100 mg); d-Alpha-tocopherol (0.225 mg); Dosage: firocoxib placebo (containing starch only); and PCSO-524®: 4 capsules/day, q24hrs PO for 4 weeks. Combination group (28 dogs); Dosage: firocoxib: 5 mg/kg, q12hrs PO; and PCSO-524®: 4 capsules/day, q24hrs PO for 4 weeks. The PCSO-524® only treatment group is not relevant to the PICO question. Force plate gait analysis to obtain peak vertical force (PVF) values: A single handler led a dog across a 10 m walkway embedded with dual force plates at a trotting pace to obtain a minimum of five valid PVF values. The limb with the smaller PVF value at week 0 was followed throughout the study as the index limb. Force plate gait analysis was performed at weeks 0, 6 (2 weeks post-treatment) and 8 (4 weeks post-treatment). OAS: The orthopaedic examination was performed by a single veterinarian who was blinded to the treatment assignment. Scores for a clinical parameter (lameness, articular pain for the hip and stifle joint) were summed. OAS was performed at weeks 0, 6 (2 weeks post-treatment) and 8 (4 weeks post-treatment). Owner questionnaire: Owners completed a canine brief pain inventory (CBPI) questionnaire (Brown et al., 2008), which contains: A severity of OA pain question set to generate a pain severity score (PSS). A question set relating to how OA pain interferes with their pet's daily activities to generate a pain interference score (PIS). The questionnaire was undertaken weeks 0, 6 (two weeks post-treatment) and 8 (4 weeks post-treatment) by owners.
Study design:	Prospective, block-randomised, double-blinded clinical trial.
Outcome studied:	PVF values. OAS. PSS. PIS.
Main Findings (relevant to PICO question)	PVF results: No significant effects between treatment groups. Significant effects of time were found within treatment groups: When comparing week 6 to week 0, PVF values for firocoxib group and combination group were significantly greater with mean ± SD change 03 ± 0.67% body weight (BW) and 2.74 ± 4.41% BW, respectively (p < 0.05 for both). When comparing week 8 to week 0, PVF values for firocoxib group and combination group were significantly increased (p < 0.05) with mean ± SD change 3.25 ± 4.13% BW and 4.11 ± 4.69% BW, respectively (p < 0.05 for both). When comparing week 6 and 8, there were no significant changes in PVF values between groups. OAS results: There were no significant treatment effects within and between treatment groups. Subjective CBPI questionnaire There were no significant treatment effects for PSS and PIS Significant effects of time were found for PIS: When comparing week 8 to week 0, PIS was significantly lower in the firocoxib group (p < 0.05). Three dogs were lost to follow up due to cranial cruciate ligament rupture (n=1), myasthenia gravis (n=1) and vehicular accident (n=1).
Limitations:	Study period (treatment duration = 4 weeks) may have been too short to detect significant differences (Zawadzki et al., 2013). The number of patients in firocoxib group was less than the calculated sample size. Inadequate sample sizes can result in false-negative results due to insufficient power to detect real differences between treatment groups (Sargeant et al., 2014). Details regarding the classification of dogs into mild/moderate or severe based on the severity of OA is unclear. Losses to follow-up was not accounted for in the sample size calculation. Whether allocation concealment was performed to prevent selection bias was not reported (this may be acceptable as this is rarely reported in veterinary trials) (Sargeant et al., 2014). There were intrinsic differences in caregiving between treatment groups, such as the conditions in which the dogs were managed during the study period, resulting in bias. PVF, OAS, PSS and PIS are surrogate outcomes and may not accurately reflect if the patient's mobility has improved or pain has reduced (Administration USFaD, n.d.).

## Appraisal, application and reflection

Osteoarthritis is a prevalent condition in dogs, with reports of up to 20% of all dogs over 1 year old, in North America affected (Anderson et al., 2020). While dogs are typically managed with a multimodal approach involving weight control, exercise moderation and analgesics/anti-inflammatories such as NSAIDs; (Anderson et al., 2020; and Belshaw et al., 2016) nutraceuticals such n-3 FAs supplements are marketed as an adjunctive therapy to NSAIDs, for both humans and companion animals (Beale, 2004; and Johnson et al., 2020). PCSO-524® is a source of n-3 FAs derived from the New Zealand green-lipped mussel, *Perna canaliculus *(Kean et al., 2013). It is reported that ingested long-chain n-3 polyunsaturated fatty acids (PUFAs) are incorporated into inflammatory cell phospholipids resulting in a decrease in the amount of arachidonic acid available for the production of AA-derived eicosanoids, such as inflammatory prostaglandins (Calder, 2009). Additionally, n-3 PUFAs may influence inflammatory cytokine production and transcription factors that regulate inflammatory gene expression (Calder, 2009). NSAID therapy is considered a mainstay therapy in the management of canine OA (Innes et al., 2010), and the efficacy of NSAID supplementation with n-3 FAs for the treatment of canine OA does have a theoretical justification. 

It is noteworthy that despite a wide search strategy, only two studies were applicable to this PICO. Both studies used block classification for the severity of OA to ensure even distribution of mild/moderate and severe OA amongst treatment groups adapted from Moreau et al. (2003).  As the difference in OA severity is a source of variation between patients, blocking aims to remove some of this variability to emphasise treatment effects (Krzywinski & Altman, 2014). However, blocking partitions the number of subjects and as the number of treatment groups increases, more subjects are required. Blocking may also interfere with maintaining homogeneity between the treatment groups (Casler, 2018).  Classification of OA severity as either mild/moderate or severe as undertaken in these studies provides some additional areas of concern. Firstly, this scoring system is not validated (Moreau et al., 2003). Secondly, the OAS was performed by a single veterinarian in Vijarnsorn et al. (2019), and it is unclear whether the OAS was performed by a single veterinarian or multiple evaluators in Kwananocha et al. (2016).  Thirdly, the OAS has many subjective parameters, such as assessment of articular pain for the affected joints. The subjective assessment of articular pain can be problematic as it is reported that variability exists between veterinarians in their perception of the level of pain in their canine patients (Gruen et al., 2020).

Randomised allocation of study subjects into treatment groups was performed in both studies. However, the method of randomisation was not reported in Kwananocha et al. (2016). As randomisation reduces selection bias during the assignment of treatments (Pannucci & Wilkins, 2010), the lack of description on the manner of randomisation may cast doubt on the experimental design rigor. To evaluate whether the groups were homogenous before the intervention, the studies undertook statistical analysis of parameters (such as body weight, age, body condition score and breed) between groups. This degree of analysis is discouraged by the CONSORT (Consolidated Standards of Reporting Trials) Statement as it interferes with proper randomisation within treatment groups (de Boer et al., 2015).

Force plate gait analysis is considered the current gold standard measure of limb function in humans and animals (Brown et al., 2013). Compared to subjective scoring such as visual observation, it is more sensitive for lameness evaluation (Quinn et al., 2007). Both studies evaluated peak vertical force (PVF) values generated via force plate gait analysis as an outcome. In bilaterally lame dogs, the index limb (to be followed throughout the study) was elected as the limb with the smaller PVF at week 0 in Vijarnsorn et al. (2019), but it was unclear what criteria was used by Kwananocha et al. (2016). Additionally, peak PVF values and OAS are only surrogate indicators and may not accurately reflect improvements in a patient's mobility or whether pain has reduced due to the treatment alone (Administration USFaD, n.d.).

While repeated measurement analysis of PVF values was performed to determine the statistical significance of treatment effects and the effect of time within treatment, only descriptive analyses (i.e. numerical PVF mean values ± SD) were provided for the change in PVF, and no further statistical analyses were reported in both studies (Kwananocha et al., 2016; and Vijarnsorn et al., 2019). The results provided in both Kwananocha et al. (2016) Table 3 and Vijarnsorn et al. (2019) Table 2 must be scrutinised carefully as a greater numerical change from day 28 post-treatment to Day 0 PVF value is seen in the combination groups. However, there is no reporting of between group analysis over this time period and consequently no further conclusions can be made, other than within groups.

There was no determination of any changes of physiological parameters within the plasma and so there was no evidence that the 3-n FAs were absorbed from the gastrointestinal tract. Furthermore, the dosage and duration of treatment was possibly insufficient for n-3FAs to reach adequate therapeutic levels. The anti-inflammatory effects of n-3 FAS are reported dose dependent (Calder, 2009) and studies in humans report a daily dosage three times greater than that used in these canine studies (Dangardt et al., 2010; and Root et al., 2013). Another study suggested that a minimum of 70 days of treatment may be required for glucosamine hydrochloride and chondroitin sulphate to take effect in osteoarthritic dogs (McCarthy et al., 2013). Additionally, n-3FAs were administered daily for 4 weeks to humans and no physiological markers in response to n-3FAs administration were detected (Root et al., 2013). However, when n-3 FAs were administered for 12 weeks to humans, cytokine tumour necrosis factor alpha (TNF-a), interleukin (IL)-1β and IL-6 had significantly decreased (p = 0.008, 0.023 and 0.035, respectively) compared to the placebo treatment group (Dangardt et al., 2010). 

Sample size calculation was performed and reported in Vijarnsorn et al. (2019), but the firocoxib only group did not meet the calculated group size. The insufficient sample size is limiting as it reduces statistical power, which lowers the chance of detecting true treatment effects (Button et al., 2013). Additionally, the investigators did not account for attrition in their sample size calculation, which is 3.7% in Vijarnsorn et al. (2019). This proportion of loss of follow-up is not a concerning source of bias for Vijarnsorn et al. (2019); as it has been suggested that losses between 5–20% may confer bias (Sargeant et al., 2014). Another reason why a larger sample size would be appropriate is to reduce the effect of confounders. This is especially pertinent in Kwananocha et al. (2016), wherein body condition score is a possible confounder and may have biased the results.

There are evident flaws in the study design in both studies, such as lack of statistical analyses on the mean changes in PVF values rather than the baseline values, short treatment period, and small sample size. Additionally, the lack of clarity regarding the randomisation process, absence of sample size calculation and unclear blocking protocols further limits the validity of the results that can be extrapolated from one publication (Kwananocha et al., 2016). While the more recent study resolves some of these shortcomings, systematic faults are still present, hindering drawing definitive conclusions about the benefits of n-3 FAs supplementation in conjunction with NSAID therapy.

The conclusion of this Knowledge Summary does not align with the conclusions in each study. Kwananocha et al. (2016) states*: The preliminary results imply the clinical benefits of PCSO-524*®* in combination with carprofen in the treatment of OA* (Kwananocha et al., 2016). Vijarnsorn et al. (2019) states: *The results of this study suggested combination of both PCSO-524*®* and firocoxib is more effective in alleviation of inflammation and improvement of weight bearing ability when compared to the uses of either PCSO-524*®* or firocoxib alone *(Vijarnsorn et al., 2019)*. *Both studies were financially supported by the supplier of the PCS0-524®, which may account for the more optimistic conclusions of both studies. A correction has since been published (Vijarnsorn et al., 2020) that the competing interests of one of the authors were omitted from Vijarnsorn et al. (2019).

## Methodology Section

**Search Strategy:** 

Databases searched and dates covered:	CAB Abstracts via Web of Science (1910—2021) Scopus (1970–present) Medline via OvidSP (1946—2021)
Search strategy:	(dog or dogs or canine or canines or canis or canid or canids or Canidae) AND (osteoarthritis or osteo-arthritis or arthritis or "joint disease" or "joint diseases" or DJD) AND (carprofen or rimadyl or rimifin or canidryl or "carprodyl F" or dolagis or rycarfa or zenecarp or carprogesic or firocoxib or previcox or NSAID or "non-steroidal" or non-steroidal) AND ("omega 3" or "omega-3" or "omega 3 oil" or "omega-3 oil" or DHA or EPA or "eicosapentaenoic acid" or "docosahexaenoic acid" or "Hexadecatrienoic acid" or HTA or "α-Linolenic acid" or "Stearidonic acid" or "Eicosatrienoic acid" or "Eicosatetraenoic acid" or "Heneicosapentaenoic acid" or "Docosapentaenoic acid" or "Clupanodonic acid" or "Tetracosapentaenoic acid" or "Tetracosahexaenoic acid" or "Nisinic acid" or ALA or SDA or ETE or ETA or HPA or DPA or "green lipped mussel" or "green-lipped mussel" or mussel or GLM or "PCSO-524" or "PCSO 524")
Dates searches performed:	15 Jul 2021

**Exclusion / Inclusion Criteria:** 

Exclusion:	Systematic reviews, narrative reviews, *in vitro* studies, conference papers, book chapters.
Inclusion:	Articles relevant to the PICO question, randomised controlled trials.

**Search Outcome:** 

Database	Number of results	Excluded – Irrelevant to PICO questions	Excluded – Systematic review	Excluded – Narrative review	Excluded – In vitro study	Excluded – Conference paper	Excluded – Book chapter	Total relevant papers
CAB Abstracts	27	10	2	8	0	6	0	1
Scopus	28	11	3	10	1	0	1	1
Medline	12	6	1	1	3	0	0	1
Total relevant papers when duplicates removed								2

N.B A correction to Vijarnsorn et al. (2019) has been published (Vijarnsorn et al. 2020)

## Conflict of Interest

The authors declare no conflicts of interest.

## Author contribution:

Lok Yee Stephanie Wong (LYSW) wrote the first draft of the manuscript with feedback from Merran Govendir (MG). LYSW and MG edited the final manuscript and undertook the emendations.

## Disclaimer

Knowledge Summaries are a peer-reviewed article type which aims to answer a clinical question based on the best available current evidence. It does not override the responsibility of the practitioner. Informed decisions should be made by considering such factors as individual clinical expertise and judgement along with patient’s circumstances and owners’ values. Knowledge Summaries are a resource to help inform and any opinions expressed within the Knowledge Summaries are the author’s own and do not necessarily reflect the view of the RCVS Knowledge. Authors are responsible for the accuracy of the content. While the Editor and Publisher believe that all content herein are in accord with current recommendations and practice at the time of publication, they accept no legal responsibility for any errors or omissions, and make no warranty, express or implied, with respect to material contained within. For further information please refer to our Terms of Use.
